# METTL3-mediated m6A methylation regulates ovarian cancer progression by recruiting myeloid-derived suppressor cells

**DOI:** 10.1186/s13578-023-01149-6

**Published:** 2023-11-06

**Authors:** Jinyong Wang, Dakai Ling, Lulin Shi, Huayun Li, Minhua Peng, Huihong Wen, Tao Liu, Ruifang Liang, Yongjian Lin, Laiyou Wei, Guangzhi Zhang, Shanze Chen

**Affiliations:** 1 Department of Pulmonary and Critical Care Medicine, Institute of Respiratory Diseases, The Second Clinical Medical College, Shenzhen People’s Hospital, Jinan University, The First Affiliated Hospital of Southern University of Science and Technology), Shenzhen, Guangdong 518020 China; 2 Institute of Infectious Diseases, Shenzhen Bay Laboratory, Shenzhen, Guangdong 518107 China; 3 Shenzhen International Institute for Biomedical Research, 518110 Shenzhen, Guangdong China; 4 Department of Hepatobiliary and Pancreas Surgery, Shenzhen People’s Hospital, Shenzhen, Guangdong 518020 China; 5 Annoroad Gene Technology Corporation, Beijing, 100176 China; 6 School of Basic Medical Sciences, Guangzhou Medical University, Guangzhou, Guangdong 511436 China; 7 Institute of Animal Sciences of Chinese Academy of Agriculture Sciences, Beijing, 100193 China; 8 Department of Microbiology and Immunology, Western University, London, ON N6A 3K7 Canada

**Keywords:** METTL3, Myeloid-derived suppressive cells, Tumor microenvironment, Inflammatory responses, Ovarian cancer, m6A

## Abstract

**Background:**

Ovarian cancer (OC) typically develops an immunosuppressive microenvironment by funtional changes of host immune cells. Dysregulated m6A level is associated with cancer progression *via* the intrinsic oncogenic pathways. However, the role of m6A in regulating host immune cell function during anti-tumor immunity needs comprehensive analysis. This study aimed to investigate the role of METTL3, a catalytic subunit of the methyltransferase complex, in regulating host immune cell response against OC.

**Methods:**

In this study, myeloid-specific *Mettl3* gene knockout (Mettl3-cKO) mice were bred using the Cre-LoxP system. Intraperitoneally injection of ID8 cells was used as a syngeneic OC model. Furthermore, the compositions of immune cell populations were analyzed by flow cytometry and single-cell sequencing. Moreover, chemokines and cytokines secretion were assessed using ELISA. Lastly, the role of METTL3 in regulating IL-1β secretion and inflammasome activation in bone marrow-derived macrophages cocultured with ID8 cells was specified by ELISA and immunoblotting.

**Results:**

It was revealed that OC cell growth was enhanced in Mettl3-cKO mice. Furthermore, a shift of decreased M1 to increased M2 macrophage polarization was observed during OC progression. Moreover, *Mettl3* depletion in myeloid lineage cells increased secretion of CCL2 and CXCL2 in peritoneal lavage fluild. Interestingly, *Mettl3* deficiency enhanced IL-1β secretion induced by viable ID8 cells independent of inflammasome activation and cell death. Therefore, OC cells in tumor-bearing mice trigger a slight inflammatory response with a low-to-moderate secretion of pro-inflammatory cytokines and chemokines.

**Conclusion:**

This study provides new insights into METTL3-mediated m6A methylation, which regulates host immune response against OC.

**Supplementary Information:**

The online version contains supplementary material available at 10.1186/s13578-023-01149-6.

## Introduction

Ovarian cancer (OC) is the most malignant and highly mortal gynecological malignancy that can be divided into subtypes based on distinct histopathology. Unfortunately, early OC development is frequently asymptomatic and lacks effective detection methods. Therefore, most patients are diagnosed at late stages, such as stages III and IV [1]. Consequently, this significantly limits the therapeutic potential while increasing the possibility of metastasis. Furthermore, OC patients frequently experience recurrence with first-line chemotherapy, reducing the overall survival rate. Therefore, a better understanding of OC tumorigenesis, particularly of the immune response, is urgently required for more effective cancer therapies.

Ascites, especially malignant ascites, are a hallmark of OC. It is a special tumor microenvironment (TME) liquid comprising various cellular and acellular components contributing to the immunosuppressive milieu, significantly promoting growth and metastasis. As major cellular components, immune cell populations infiltrating the TME have various pro- or anti-tumor activities. Among these cells, myeloid-derived suppressive cells (MDSCs) play a critical role in tumor immunity. They are heterogeneous populations of immature and mature myeloid cells, including dendritic cells, granulocytes, and macrophage precursors [2]. MDSCs are crucial in developing cancer, non-cancerous chronic inflammations, and autoimmune diseases. For example, deleting myeloid cells with anti-CD11b antibody delayed tumor progression in a metastatic syngeneic mouse model of epithelial OC [3]. In addition, γ-δ T cells promote tumor growth of OC by secreting IL-17 A and mobilizing pro-tumor peritoneal macrophages (PMs) through the IL-17 A/IL-17R α axis [4]. The PMs are heterogeneous in size, function, and development [5] and are categorized as small PMs (SPMs) and large PMs (LPMs) subsets. PMs can be switched to tumor-associated macrophages (TAMs) in ascites with pro-tumor effect by releasing various soluble mediators, including IL-6, IL-10, CCL18, CCL22, TNF-α, TGF-β, and EGF that triggers pro-tumorigenic signaling pathways in tumor cells and infiltrating leukocytes in the TME [6–8]. Moreover, it is suggested that TAMs and tumor cells can cooperate in extracellular matrix remodeling, a prerequisite for tumor cell adhesion and invasion [9]. Overall, TAMs create an immunosuppressive microenvironment, thereby promoting tumor growth [8] and metastasis of OC [10].

Trans-coelomic metastasis is a major and unique metastatic route of OC, during which tumor cells detach from the primary tumor site and migrate within the peritoneal cavity (PerC). The peritoneum is among the preferred metastatic sites of tumor cells, where TAMs support their survival [8]. Additionally, it has been proved that tissue-resident macrophages in the omentum [10] and infiltrating neutrophils [11] can promote OC metastasis and contribute to a premetastatic niche during OC progression. Recently, a study indicated that cancer-associated fibroblasts are important for spheroids (cell aggregates) formation, amplifying the tumor-stroma interaction and consequently promoting trans-coelomic metastasis of OC [12]. Therefore, elucidating the interactions between immune and tumor cells is crucial for understanding tumorigenesis and metastasis of OC.

In nearly all eukaryotes, N6-methyladenosine (m6A) is a highly prevalent reversible chemical modification in mRNA and non-coding RNAs, affecting RNA splicing, translation, and stability [13]. m6A is regulated by a dynamic interaction of protein complex consisting of methyltransferases, demethylases, and binding proteins, which are known as “writers,“ “erasers,“ and “readers,“ respectively [14]. METTL3, a catalytic subunit of the methyltransferase complex, has been reported to contribute to various biological processes, including spermatogenesis [15], neurogenesis [16–18], stem cell self-renewal [19–22], T cell homeostasis [23], follicular T cell differentiation [24], B cells development [25], and immunoregulation [26, 27]. Moreover, it has been demonstrated that dysregulated m6A levels influence the intrinsic oncogenic pathways of tumor cells, thereby promoting the progression of various cancers [28–32]. However, its potential roles in the host immune cells and anti-tumor immune response remain to be determined.

This study revealed that myeloid-specific deletion of *Mettl3* enhanced OC in a syngeneic mouse OC model. Furthermore, the *Mettl3* deficiency in myeloid lineage cells increased the recruitment of Gr-1^+^ MDSCs during OC progression. This study provides new insights into the regulation of host immune response against OC *via* m6A.

## Results

### Generation of Mettl3-cKO mice and ID8 syngeneic mouse model

To investigate the role of METTL3 in the immune response against OC, the myeloid-specific *Mettl3* gene knockout mice were generated by using the Cre-LoxP system in which *Mettl3*^*fl/fl*^ mice were crossed with *Lyz2-Cre*^*+/+*^ mice. The offspring were genotyped, and female mice at 6–8 weeks were selected for subsequent analyses. The conditional *Mettl3* knockout efficiency in *Mettl3*^*fl/fl*^*Lyz2-Cre*^*+/+*^ (Mettl3-cKO) was verified using bone marrow-derived macrophages (BMDMs) with the help of quantitative real-time PCR (qRT-PCR) and Western blot (Fig. S1-A, S1-B). The m6A status of BMDM was verified by dot blot using anti-m6A antibody (Fig. S1-C).

ID8 is a well-characterized cell line frequently used as a syngeneic mouse model for epithelial OC. To verify the tumorigenicity of these cells, 1 × 10^5^ and 5 × 10^5^ cells were intraperitoneally injected into WT mice and then monitored for eight weeks. The results showed that both doses of ID8 cells yielded robust tumor cell growth, as indicated by the formation of ascites and spheroids in the PerC of WT mice (Fig. S2-A, S2-B). Eight weeks after the injection, mice who received the higher dose (5 × 10^5^) of ID8 cells indicated significantly bloody ascites in the PerC, which further supports the characteristics of the ID8 cell line as a representative syngeneic mouse model for OC. Since OC cells are liquid tumors during the early stage of trans-coelomic metastasis, detached OC cells must receive necessary matrix support to overcome anoikis. Consistent with this and a previous report [8], the proximity of PMs (CD68^+^) and ID8 cells (Ki-67^+^) within the spheroids was observed after eight weeks of implantation (Fig. S2-C). These results demonstrated that a syngeneic mouse model of OC was successfully established using the ID8 cell line.

### Ovarian cancer cell growth was enhanced in mice with myeloid-specific deletion of *Mettl3*

To explore whether myeloid-specific *Mettl3* deletion affects ID8 cell growth in vivo, 5 × 10^5^ cells were intraperioneally injected in WT or Mettl3-cKO mice. Furthermore, ID8 cell proliferation, spheroids formation, ascites formation, and total peritoneal cells (total PerC) were measured and compared at different time points in these mice. A previously reported surface marker, CD45, was used to differentiate host pan-hematopoietic (CD45^+^) and ID8 tumor cells (CD45^−^) [33].

**Fig. 1:**
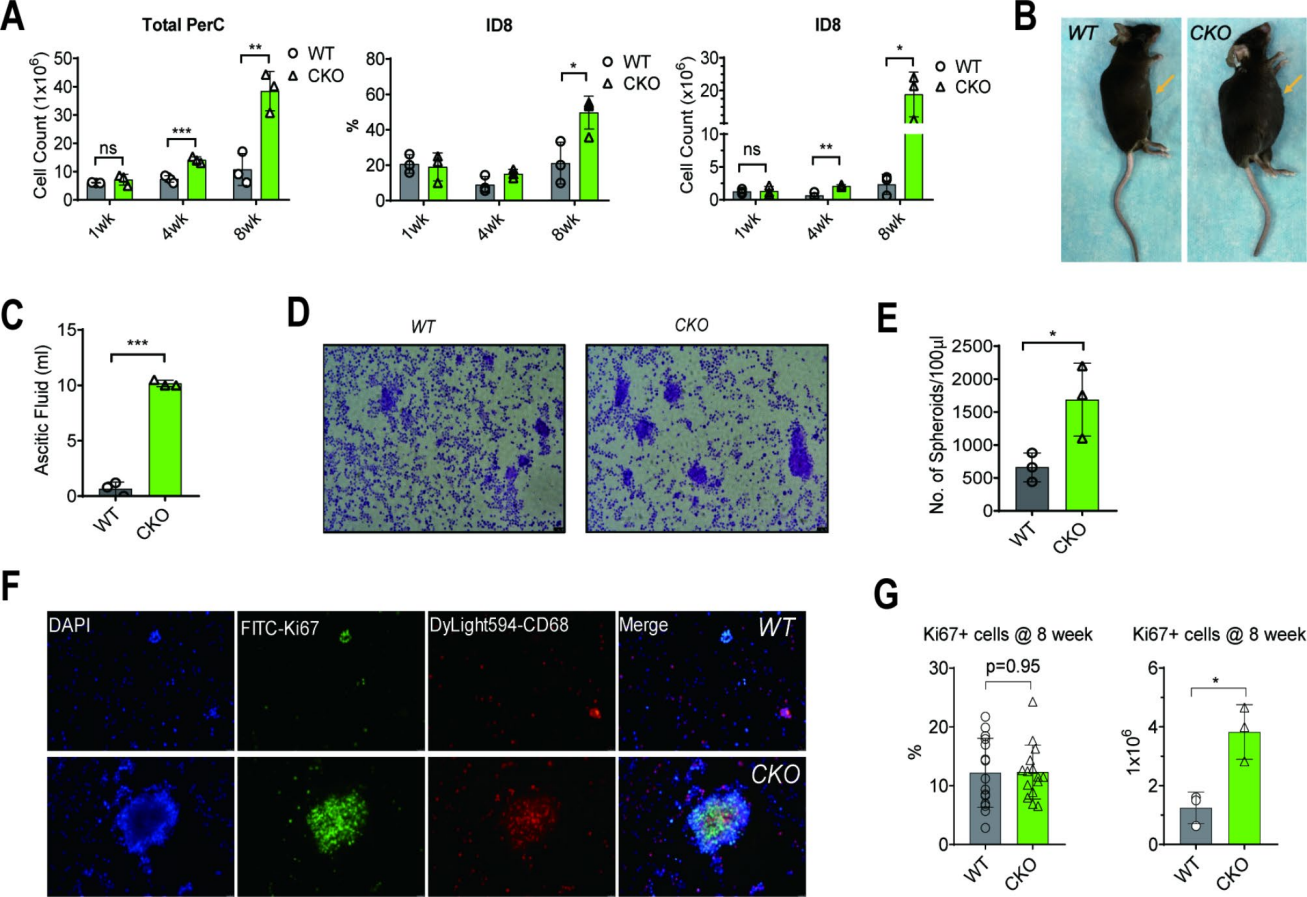
ID8 cell growth in WT and Mettl3-cKO mice. (**A**) 5 × 10^5^ ID8 cells were intraperitoneally injected into WT and Mettl3-cKO mice. Mice were sacrificed at 1, 4, and 8 weeks. Total PerC cells were obtained for each mouse by pooling ascites (if present) and peritoneal lavage. The percentage of ID8 (live CD45^−^) cells was analyzed by flow cytometry, and the ID8 cell count was assessed based on the total cell count. Each dot represents one animal. Bars represent SD; *p < 0.05, **p < 0.01, ***p < 0.001. (**B**) Representative images of mice abdomen indicating severe ascites formation in Mettl3-cKO mice at week 8. (**C**) The volume of ascites collected from WT and Mettl3-cKO mice at week 8. Each dot represents one animal. Bars represent SD; ***p < 0.001. (**D**) Representative H&E staining images of total PerC cells indicate that tumor-bearing Mettl3-cKO mice had more and larger spheroids than WT mice. (**E**) The numbers of spheroids are normalized to each 100 µL ascites from WT and Mettl3-cKO mice at week 8. Each dot represents one animal. Bars represent SD; *p < 0.05. (**F**) The immunofluorescent staining assay confirms a close interaction between PMs and ID8 tumor cells. PMs: CD68^+^; ID8 cells: Ki67^+^. (**G**) Percentages and total numbers of Ki67^+^ cells, indicating ID8 tumor cells, were compared between tumor-bearing WT and Mettl3-cKO mice at week 8. Each dot represents a cell count in one field. Bars represent SD; ns, *p* > 0.05, *p < 0.05

It was revealed that ascites gradually accumulated with increased total cells in the PerC. Furthermore, in Mettl3-cKO mice, ID8 cell growth was markedly enhanced at a later time point, eight weeks post-implantation, than that in WT mice (Fig. 1A, Fig. S3C). The enhanced tumor growth in Mettl3-cKO mice coincided with accelerated ascites formation presented by a remarkably expanded abdomen (Fig. 1B and Fig. S3-A) and an increased volume of bloody ascites (Fig. 1C, Fig. S3-B). Consistently, Mettl3-cKO mice also indicated substantially increased size and number of spheroid formations compared with WT mice (Fig. 1D-E). Although ID8 growth was enhanced in Mettl3-cKO mice, the percentages of Ki67^+^ ID8 cells in total PerC cells were comparable between Mettl3-cKO and WT mice (Fig. 1F-G), indicating the proliferative capacity of ID8 cells was not altered under certain circumstances. These results suggest that the TME immune response and not the ID8 cell’s proliferative capacity account for enhanced tumor cell growth in Mettl3-cKO mice.

### *Mettl3* deficiency in macrophages minimally alters composition but not polarization of peritoneal SPMs and LPMs

**Fig. 2:**
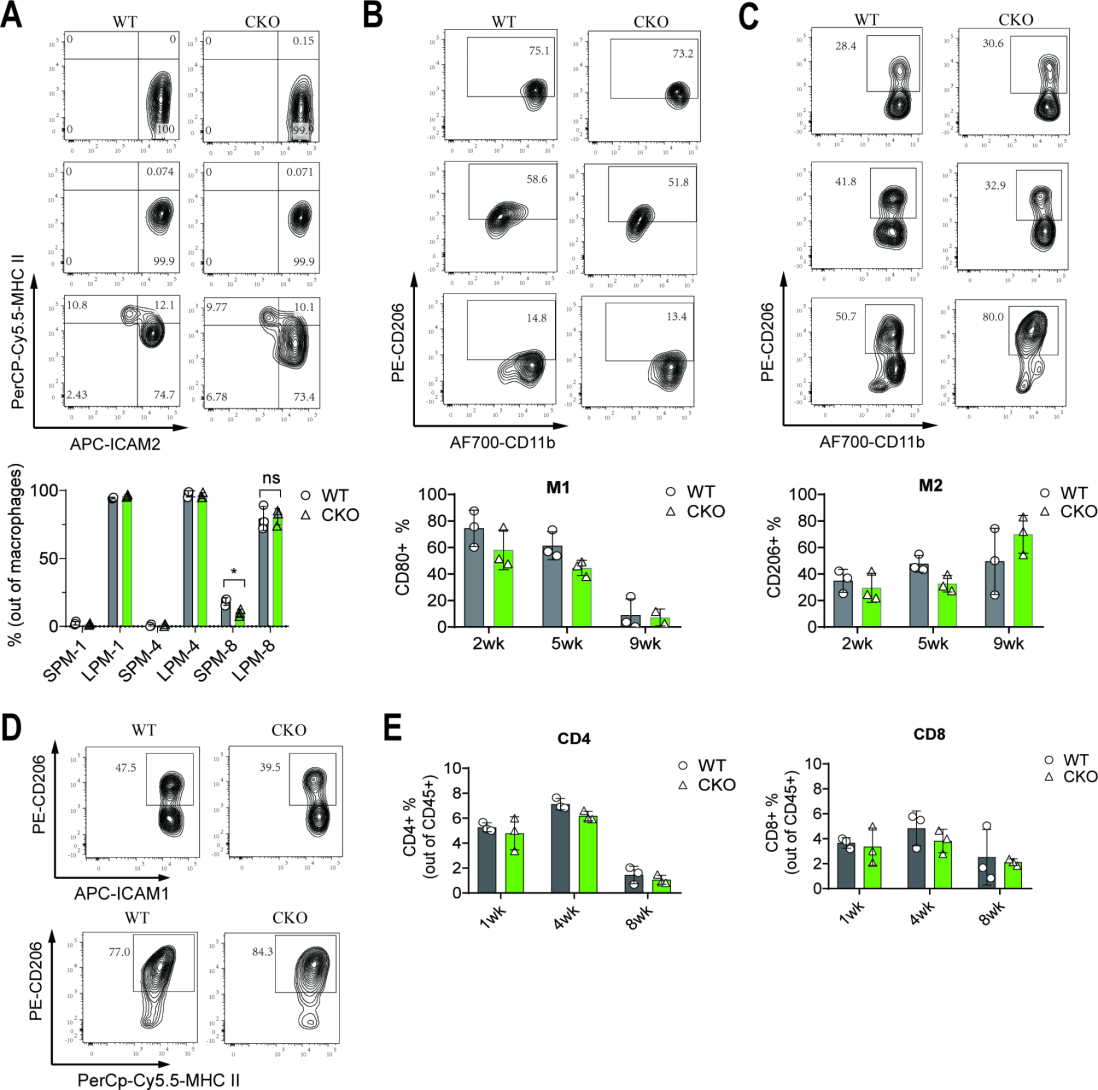
*Mettl3* deficiency in macrophages alters the composition and not polarization of peritoneal SPMs and LPMs. The total PerC cells were collected and analyzed with flow cytometry. (**A**) Representative FACS plots and percentage of SPMs and LPMs at 1, 4, and 8 weeks indicated a slight increase of SPMs but decreased LPMs in WT and Mettl3-cKO mice throughout tumorigenesis. (**B-C**) Representative FACS plots of M1 and M2 subsets (based on CD11b expression) at weeks 2, 5, and 9. (**D**) Representative FACS plots of M2 differentation of ICAM-1^+^ SPM and MHC-II^+^ LPM. The percentage of two macrophage subsets indicated similar M1 and M2 polarization between WT and Mettl3-cKO mice. (**E**) The percentage of CD4 + T and CD8 + T cells revealed no discrepancy between WT and Mettl3-cKO mice. Each dot represents one animal. Bars represent SD.

The compositions of different immune cell populations in the TME were assessed by flow cytometry. First, the residing and infiltrating macrophages in the PerC (tissue-resident and monocytes-derived macrophages, respectively) were analyzed, which revealed that the SPM (CD11b^+^ICAM-1^+^MHC-II^−^) accumulation tendency slowly increased after ID8 cells implantation (Fig. 2A). In WT mice, the percentage of SPMs in PerC was retained at a basic level (< 5%) until four weeks and significantly increased to 15–20% at eight weeks after ID8 cell implantation. Furthermore, it was revealed that the SPM accumulation was significantly slower in Mettl3-cKO mice than in WT mice, indicating that the TME differs. However, the percentage of LPMs (CD11b^+^ICAM-1^−^MHC-II^+^) in PerC remained unchanged until four weeks and significantly decreased (from 95% to 70–85%) at eight weeks. These results suggest that ID8 cells only induce a moderate inflammatory response with a slight increase of SPMs in the late phase of tumorigenesis associated with METTL3.

Previous in vitro and in vivo studies have suggested that OC cells induce macrophage polarization toward TAMs [34, 35], which have an M2 phenotype closely related to tumorigenesis and can modulate the TME by releasing various cytokines, chemokines, and metabolites [36]. Therefore, the M1/M2 phenotype of PMs was differentiated by CD80 (for M1) and CD206 (for M2), which indicated that the initial polarization (at two weeks) of PMs was dominated by an anti-tumor M1 phenotype (Fig. 2B-C) and as the tumor progressed, PMs gradually polarized toward an M2 phenotype, with only a small M1 macrophages proportion at the late stages (nine weeks) (Fig. 2B-C). However, the PMs subpopulation (SPM and LPM, Fig. 2D) from both WT and Mettl3-cKO mice had similar M1/M2 phenotyping during tumor progression (two to nine weeks), indicating that METTL3 is not involved in the M1 to M2 polarization during OC progression.

Moreover, it was assessed if *Mettl3* deficiency in macrophages affects the infiltration of T cells during OC cell progression. The literature suggests that *Mettl3* inhibition in macrophages promotes Treg cells in the TME [37]. This study showed that CD4^+^ and CD8^+^ T cells had steadily maintained levels without significant change throughout OC progression (one to eight weeks) (Fig. 2E). It collectively suggests that *Mettl3* deficiency alters compositions but not polarization of PMs during OC progression.

### *Mettl3* depletion in myeloid cells inceased the Gr-1^+^ MDSCs and elevated the CCL2 and CXCL2 secretion in the peritoneal cavity

**Fig. 3:**
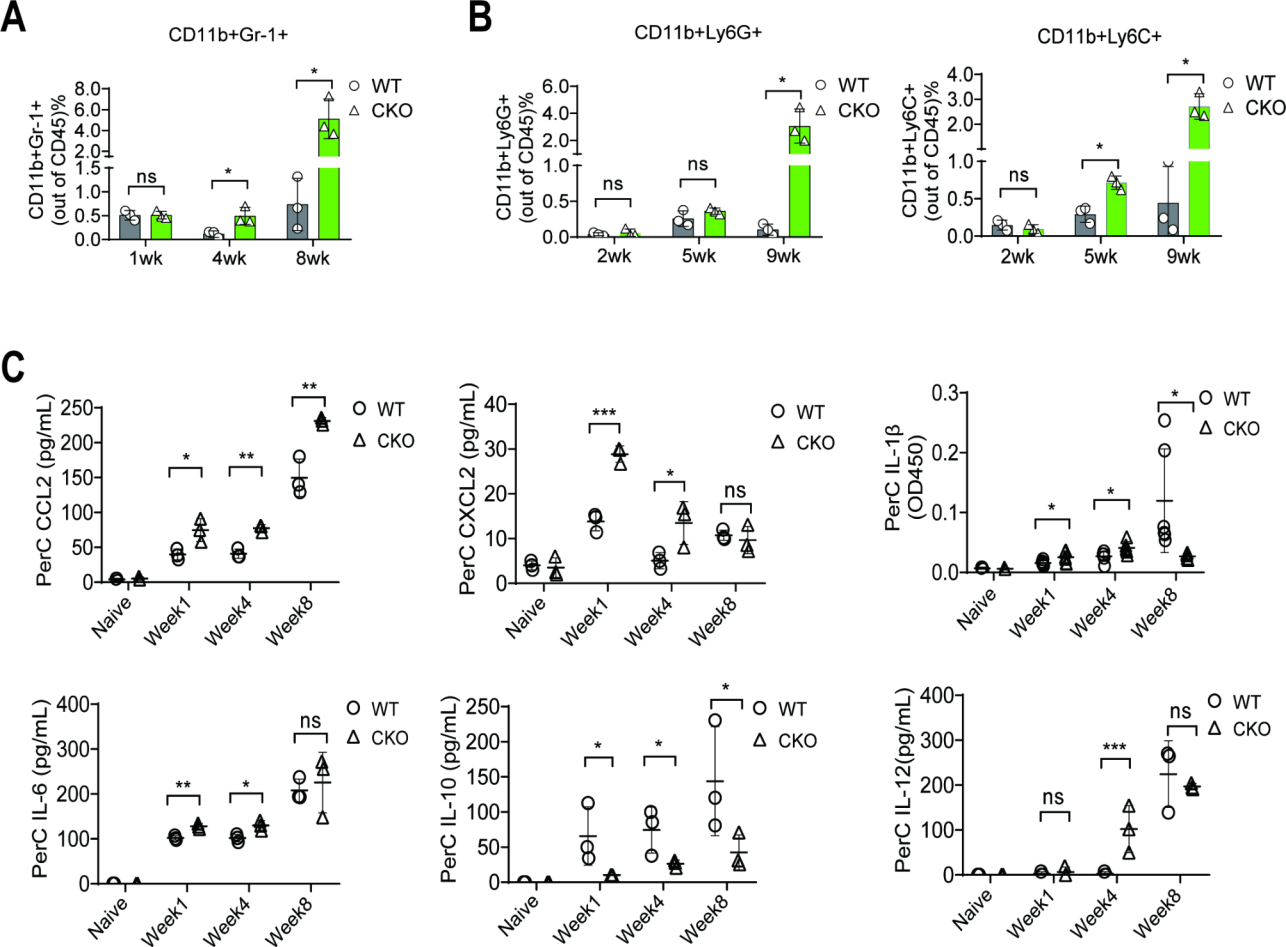
*Mettl3* deletion in myeloid cells facilitates the recruitment of Gr-1^+^ MDSCs. (**A**) Flow cytometry analysis of total PerC cells demonstrated gradual recruitment of CD11b^+^Gr-1^+^ MDSCs during the early and mid-phase ID8 tumorigenesis, and considerably increased MDSCs recruitment in the peritoneum of Mettl3-cKO mice was observed during the late stage of tumorigenesis. (**B**) MDSCs are further classified into PMN-MDSCs and M-MDSCs based on Ly6G and Ly6C expression, respectively. (**C**) Multiple cytokines in the peritoneal lavage of mice at weeks 1, 4, and 8 were analyzed with ELISA. Each dot represents one animal. Bars represent SD; *p < 0.05, **p < 0.01, ***p < 0.005

This study proposed that other immune cells, such as MDSCs, may contribute to the difference in OC progression between WT and Mettl3-cKO mice. It was observed that in Mettl3-cKO mice, Gr-1^+^ MDSCs were marginally present in PerC until four to five weeks after ID8 cell inoculation but were dramatically increased in the late phase (eight to nine weeks) of tumor progression (Fig. 3A). Further analysis of granulocytic MDSCs (PMN-MDSCs) and monocytic MDSCs (M-MDSCs) subsets, as previously reported [38, 39], revealed that their increased populations remarkably increased Gr-1^+^ MDSCs in Mettl3-cKO mice (Fig. 3B). These results suggest that the enhanced recruitment of Gr-1^+^ MDSCs into the PerC may accelerate ID8 cells growth in Mettl3-cKO mice compared with WT mice.

To investigate why Mettl3-cKO mice recruit more Gr-1^+^ MDSCs in the PerC after OC cell implantation, the secretion of various chemokines and cytokines in peritoneal lavage and serum was screened and compared by ELISA in tumor-bearing WT and Mettl3-cKO mice. CCL2 and CXCL2 are the main chemokines implicated in M-MDSC and PMN-MDSCs migration to tumors [40, 41]. Interestingly, a substantial infiltration of CCL2 and CXCL2 was observed in peritoneal lavage (Fig. 3C) and peripheral blood (Fig. S4) after ID8 cell implantation. Furthermore, compared with tumor-bearing WT mice, Mettl3-cKO mice indicated a significantly higher secretion of CCL2 in peritoneal lavage and peripheral blood and CXCL2 in only peritoneal lavage (Fig. 3C) but not in peripheral blood (Fig. S4). ELISA revealed that in Mettl3-cKO mice, IL-6 and IL-12 were increased in the peritoneal lavage, but IL-10 was decreased in peritoneal lavage and serum (Fig. 3C). Furthermore, IL-1β, a potent pro-inflammatory cytokine, was gradually induced in PerC in WT mice remained at a high level after OC implantation (Fig. 3C) whereas, in Mettl3-cKO mice, its levels were higher until four weeks, then quickly diminished at eight weeks. Thus, the data suggested that *Mettl3* is involved in MDSCs recruitment in which a complex network may mediate between different cytokines and chemokines such as IL-1β、CCL2 and CXCL2 signaling. It has been shown that IL-1β facilitates robust upregulation of CXCL1/2 and CCL2 in ID8 cells [42]. However, whether ID8 cells stimulate IL-1β secretion needs further evaluation.

### Viable ID8 cells but not cell lysates enhance IL-1β production in *Mettl3*-depleted macrophages Independent of inflammasome activation

**Fig. 4:**
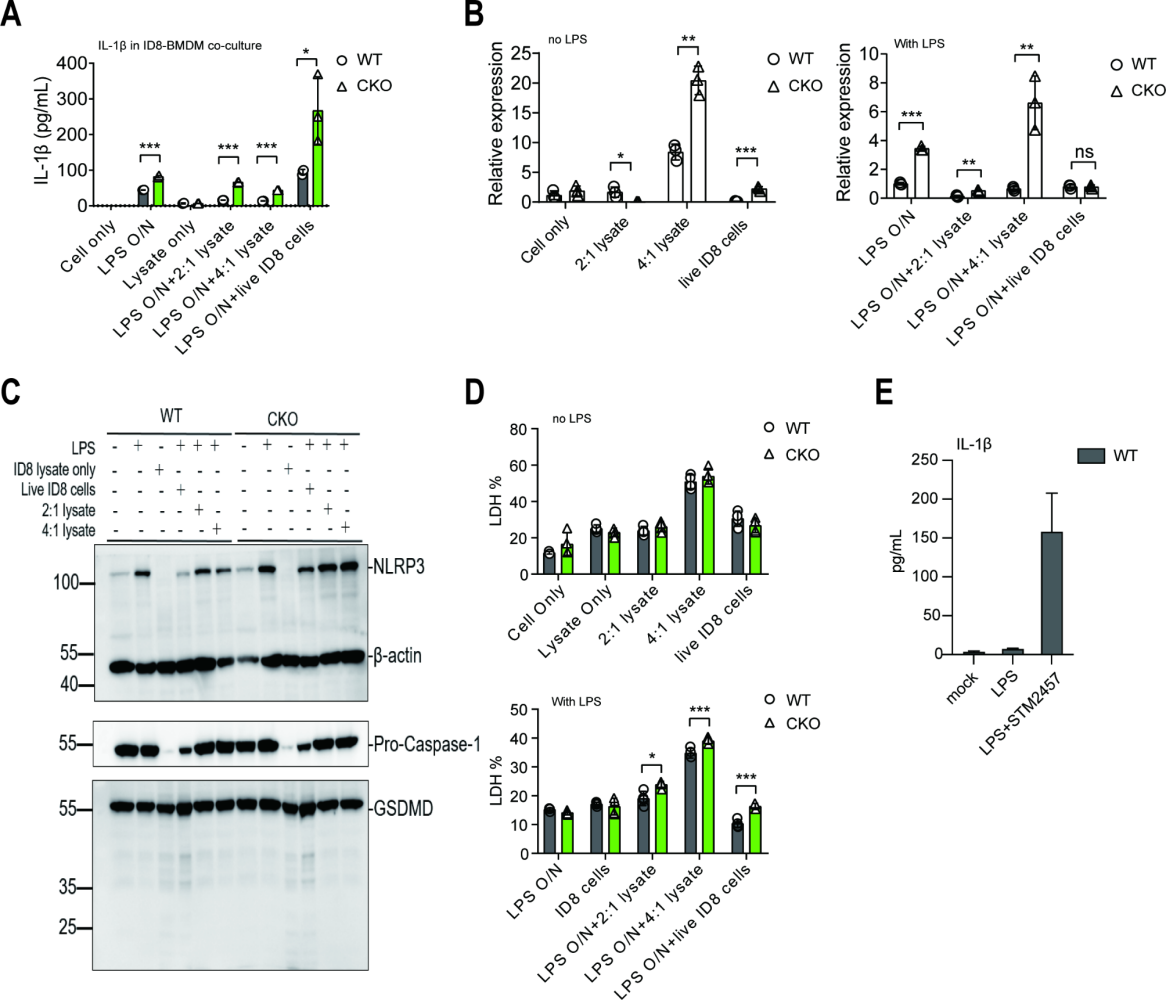
Viable ID8 cells enhance IL-1β production by *Mettl3*-deficient macrophages independent of inflammasome activation. (**A**) LPS-stimulated BMDMs were treated with viable ID8 cells or their lysate in vitro, and their supernatants were collected to measure IL-1β levels *via* ELISA. Elevated IL-1β level was measured in *Mettl3*-deficient BMDMs across all treatment groups compared with WT BMDMs. Moreover, viable ID8 cells treated with *Mettl3*-deficient BMDMs revealed increased IL-1β levels than those treated with ID8 cell lysate. (**B**) The transcriptional level of IL-1β mRNA in BMDMs treated with viable ID8 cells or ID8 cell lysate was analyzed by qRT-PCR. Treatment with ID8 cell lysate (4:1 but not 2:1) significantly elevated IL-1β mRNA transcription in *Mettl3*-deficient BMDMs than in WT BMDMs with or without LPS stimulation. ***p < 0.001. Only LPS stimulation and dead ID8 cells elicited significantly increased IL-1β mRNA transcription in *Mettl3*-deficient BMDMs than in WT BMDMs. ***p < 0.001. (**C**) Western blot analysis of inflammasome-related proteins. (**D**) Cell cytotoxicity was measured by an LDH release assay. No significant difference in cell death or cytotoxicity was observed between WT and *Mettl3*-deficient BMDMs without LPS stimulation. Data from one of six independent experiments are shown. Bars represent SD; *p < 0.05, **p < 0.01, ***p < 0.005

To specifically study the role of METTL3 in regulating IL-1β secretion, *in-vitro* BMDMs coculture experiments with viable ID8 cells or their lysates were performed. The expression level of IL-1β was measured by qRT-PCR and ELISA, which revealed two captivating outcomes (Fig. 4A and Fig. S5). First, ELISA showed that viable ID8 cells but not their lysates induced a significant increase of IL-1β secretion in cocultured supernatants. Secondly, compared to WT BMDMs, *Mettl3*-deficient BMDMs produced an extensive and much higher IL-1β secretion when cocultured with live ID8 cells after LPS pre-treatment. These data suggest that LPS priming and neither ID8 lysate nor live ID8 cells are required for a successful IL-1β secretion. Moreover, IL-1β secretion by BMDMs is accelerated in the absence of *Mettl3*, suggesting METTL3s’ regulatory role in the pro-inflammatory response. Interestingly, without LPS pre-treatment, IL-1β was not detectable in the cocultured supernatants of BMDMs (data not shown), even if ID8 cell lysates induced the transcription level of IL-1β (e.g., 4: 1 lysate) or in living ID8 cells (Fig. 4B left panel). It should be noted that no IL-1β was secreted by ID8 cells, albeit transcription of IL-1β mRNA in ID8 cells was detected (Fig. S5-B). The phenomenon of higher IL-1β secretion by BMDMs was reproduced with a specific catalytic inhibitor of Mettl3 (Fig. S5D). Altogether, these results indicate that LPS stimulation crucially regulates IL-1β secretion by BMDMs, possibly *via* a mechanism of priming cells to initiate IL-1β transcription or other genes. This leads to a hypersensitive cellular status, specifically in the absence of *Mettl3*.

Conventionally, IL-1β maturation and secretion require activated inflammasome to cleave its precursor pro-IL-1β to an active form and GSDMD N-terminal induced loss of cellular integrity, directly forms membrane pores. Next, to explore whether METTL3 regulates IL-1β by influencing inflammasome activation and cell death, Western blot and LDH release assays were performed to assess the cleavage of effector proteins and cell death, respectively. Interestingly, no inflammasome activation was observed in any tested samples (Fig. 4C), albeit a significantly higher induction of NLRP3 expression in *Mettl3*-deficient BMDMs than that in WT BMDMs. Furthermore, it was noticed that ID8 cells could express NLRP3 and caspase-1 only at a very low level or rarely detectable under static conditions. In contrast, their expression was substantially enhanced after overnight LPS treatment (Fig. 4C, Lane 3 vs. Lane 4 for WT BMDMs, Lane 9 vs. Lane 10 for *Mettl3*-deficient BMDMs).

The cultured supernatants were collected and subjected to LDH release assay (Fig. 4D and Fig. S5-C), which indicated no difference in LDH release between WT and *Mettl3*-deficient BMDMs, suggesting that enhanced IL-1β secretion in *Mettl3*-deficient BMDMs was not dependent on cell death, consistent with previous study [43] reporting that IL-1 family cytokines can be secreted without cell death. The data indicate that *Mettl3* deficiency enhances IL-1β secretion induced by viable ID8 cells but not cell lysates independent of inflammasome activation and cell death.

### Single-cell sequencing analysis confirms enhanced MDSCs recruitment in Mettl3-cKO mice after ID8 implantation

**Fig. 5:**
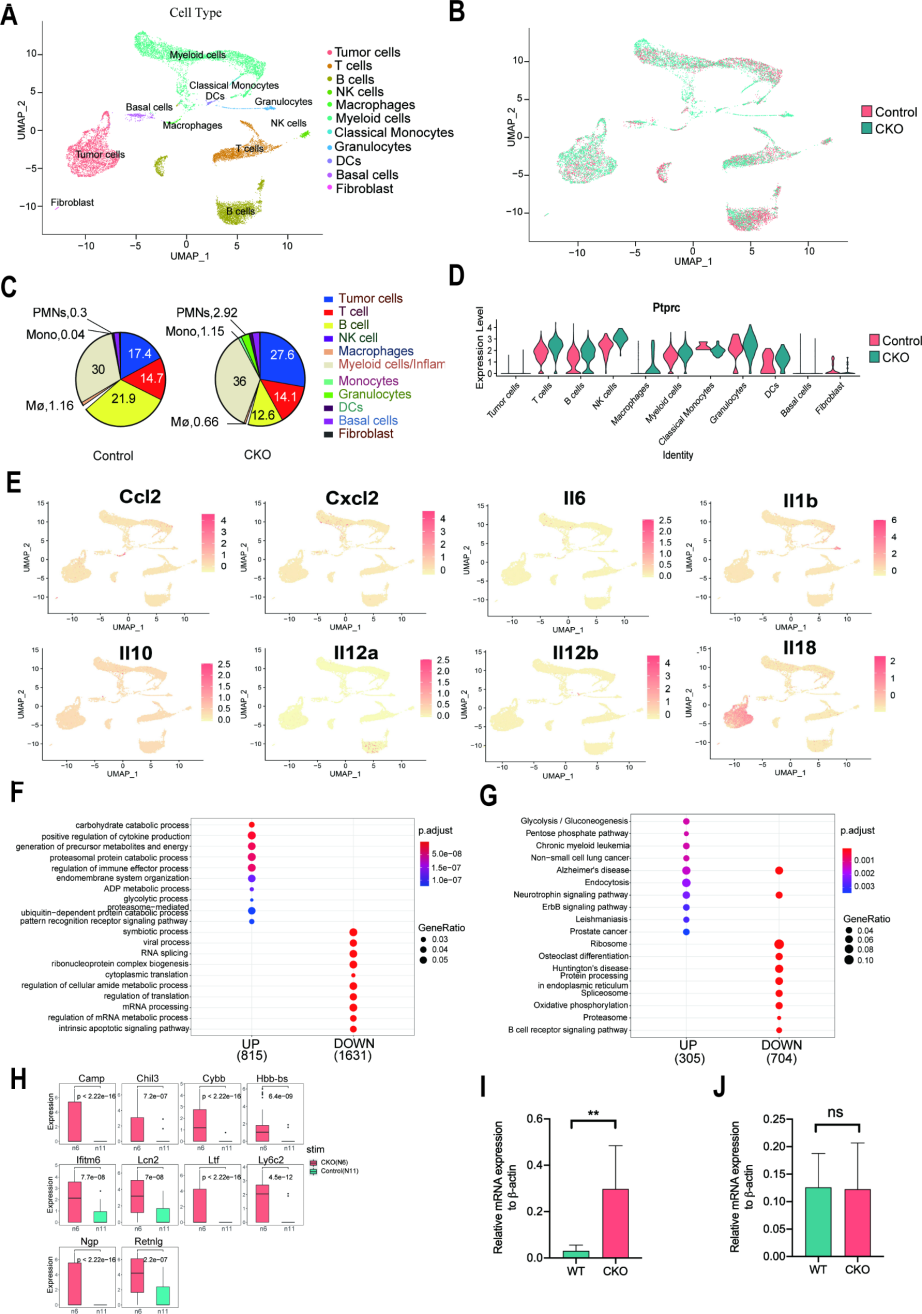
Single-cell sequencing analysis confirms enhanced MDSCs recruitment and chemokines secretion in tumor-bearing Mettl3-cKO mice. Seurat analysis eventually clustered 11 cell populations from both WT and Mettl3-cKO mice. (**A**) Integrated UMAP image of 11 major cell populations in PerC from tumor-bearing WT mice. (**B**) Merged UMAP image of two analyzed samples: PerC cells from tumor-bearing WT and Mettl3-cKO mice. (**C**) Representative proportions of distinct cell populations in WT and Mettl3-cKO mice. (**D**) The transcriptional level of CD45 mRNA (by *Ptprc*) among 24 distinct cell populations. (**E**) Transcriptional expression of different cytokines and chemokines in subpopulations at the single-cell level. (**F**) Gene Ontology Enrichment Analysis using the clusterProfiler package revealed differentially expressed genes of “Granulocyte” subpopulations between WT and Mettl3-cKO mice. A total of 815 up-regulated and 1631 down-regulated genes were identified in granulocytes from Mettl3-cKO mice compared with that in WT mice. (**G**) KEGG pathway analysis of differential expressed genes of “Granulocyte” subpopulations between WT and Mettl3-cKO mice. Size is proportional to the number of differential expressed genes. Red to blue colors represent different adjusted P values. (**H**) The top ten differential expressed genes in granulocytes of Mettl3-cKO mice are listed. (**I**) *Cybb* mRNA expression level in granulocytes isolated from tumor-bearing WT or Mettl3-cKO mice was verified by qRT-PCR. (**J**) *Cybb* mRNA expression level in granulocytes isolated from the bone marrow of WT or Mettl3-cKO mice was verified by qRT-PCR.

To confirm the impact of METTL3 on the immune response, cells acquired from peritoneal lavage of tumor-bearing WT and Mettl3-cKO mice were analyzed through single-cell RNA sequencing. The Seurat analysis identified cell clusters in three compartments based on surface markers. The tumor compartment included four clusters of OC cells (clusters 2, 8, 9, and 20). The immune compartment comprised myeloid cells (clusters 4, 5, 6, 7, 12, and 14), classical monocytes (cluster 22), T cells (clusters 3, 10, and 16), B cells (clusters 1, 11, 17, and 23), residual macrophages (cluster 19), granulocytes (cluster 15), antigen-presenting cells (cluster 21), and NK cells (cluster 18). Furthermore, the stromal compartment included basal cells (cluster 13) and fibroblasts (cluster 24) (Fig. 5A and Fig. S6). Ten host and one tumor cell clusters were identified in WT and Mettl3-cKO mice (Fig. 5B). Since the majority of ID8 cells do not express CD45 (encoded by *Ptprc*) [33], ID8 tumor cells in the sample were differentiated by the negative expression for CD45 (Fig. 5D). Subsequently, these 11 cell clusters were summarized and quantified in their sample, which revealed a higher proportion of OC cells in Mettl3-cKO mice than in WT mice (27.6% vs. 17.4%) (Fig. 5C). Meanwhile, the data showed higher proportions of polymorphonuclear neutrophils, monocytes, and inflammatory macrophages but a lower proportion of B cells in Mettl3-cKO mice than that in WT mice. The enhanced recruitment of polymorphonuclear neutrophils and monocytes was consistent with flow cytometry results (Fig. 3). Surprisingly, the single-cell sequencing data revealed reduced B cell and increased myeloid cell population in tumor-bearing Mettl3-cKO mice than that in WT mice. This difference must be investigated further if it accelerates ID8 cell growth in Mettl3-cKO mice. To validate the acquired results of cytokine secretion, the transcriptional level of corresponding cytokine or chemokine genes in different subpopulations was analyzed (Fig. 5E).

### *Mettl3* deficiency in myeloid cells increases the expression of *Cybb* in MDSCs to promote pro-tumorigenic features

Next, the functional changes of the relatively abundant granulocyte population were analyzed by investigating differentially expressed genes (DEGs) in tumor-bearing Mettl3-cKO and WT mice. A total of 815 up-regulated and 1631 downregulated genes were identified. GO analysis revealed that these DEGs were substantially related to molecular function, cellular component, and biological process (Fig. 5F). Furthermore, KEGG analysis indicated that these DEGs were mainly enriched in the ribosome, spliceosome, and protein processing in the endoplasmic reticulum (Fig. 5G). Subsequently, the top 10 markedly up-regulated genes in granulocytes from Mettl3-cKO mice were identified (Fig. 5H). Among these, *Cybb* indicated significant up-regulation in granulocytes isolated from periteoneal lavage of Mettl3-cKO mice with ID8 tumor-bearing compared with WT mice (Fig. 5I). However, *Cybb* mRNA expression displayed no difference in naive neutrophils isolated from bone marrow of WT and Mettl3-cKO mice (Fig. 5J). MDSCs impede immune-mediated malignant cell clearance by multiple mechanisms, including the formation of immunosuppressive reactive oxygen species (ROS). Furthermore, *Cybb* has been reported to be up-regulated in PMN-MDSCs [44] and generate ROS *via* NADPH oxidase (NOX2, encoded by *Cybb*). Therefore, it was hypothesized that increased NOX2 expression is an important marker in the immunosuppressive capacity of tumor-infiltrating MDSCs. Altogether, *Mettl3* deletion in myeloid cells increases *Cybb* expression, forming a tumor-promoting feature of granulocytes during immune response against tumor growth. However, the exact role of METTL3 in NOX2 expression needs further investigation.

## Discussion

The immunobiology of OC differs from that of hematological malignancy because OC cells generally originate from the following three ovarian sites: surface, fallopian tube, or mesothelium-lined PerC. Furthermore, it primarily disseminates across the PerC. The hallmark of late-metastasis of OC is the formation of ascites in the peritoneum, comprising diverse immune and tumor cells. At a steady state, macrophages in the PerC (PMs) are heterogeneous in size, function, and development [5]. Two macrophage subsets, SPMs and LPMs, were identified, and they can be further detected with the co-expression of ICAM2 and MHC-II. SPMs constitute a minor part (~ 5%) of unstimulated PMs and accumulate gradually in response to an inflammatory stimulus. Conversely, LPMs form a major part of PMs at steady-state and disappear rapidly after inflammatory stimulations.

Since PerC is a singular compartment where various immune cells can interact with OC cells during metastasis, the cellular composition in the PerC during OC progression is important to elucidate the dynamic interaction between immune and tumor cells. However, because of the complex role and classification, TAM activity during OC progression still needs further assessment. Emerging evidence indicates that circulating monocytes are not the sole and major source of tissue macrophages [44, 45]. Furthermore, embryonic-derived tissue macrophages have self-renewal capacity regulated by different physiological tissue-specific microenvironments [46, 47]. Tissue-resident macrophages are also a substantial source of the TAMs [48, 49], as revealed by genomic profile similarity between resident PMs and TAMs isolated from OC ascites. Both cell populations highly express CD63 and CD206, with genes involved in phagocytosis and antigen presentations [50]. It has been proposed that circulating monocytic- and embryonic-derived macrophages play different roles in tumor immune response. However, the markers that can be used to differentiate these two sources of macrophages remain undetermined.

Recently, a study reported that Tim-4 (T cell immunoglobulin and mucin domain-containing 4) can differentiate residential PMs in murine OC models: Tim-4^+^ TAMs that are embryonically originated and locally sustained, whereas Tim-4^−^ TAMs are replenished from circulating monocytes [51]. Tim-4^+^ TAMs but not TIM-4^−^ TAMs promoted tumor growth in vivo. This is consistent with the study classifying the two subsets of F4/80^hi^MHC-II^low^ and F4/80^low^MHC-II^hi^ [51, 52]. TAM-4^+^ TAMs represent most of the F4/80^hi^MHC-II^low^ subset, while Tim-4^−^ TAMs represent most F4/80^low^MHC-II^hi^ subset [51]. This study monitored the percentage of SPMs and LPMs during OC progression between WT and Mettl3-cKO mice. It revealed that in both mice cohorts, the SPM population slowly accumulates locally in the PerC with tumor development. In contrast, the LPM population (ICAM2^+^MHC-II^lo^) predominates the PMs until 8–9 weeks after ID8 cancer cells inoculation (Fig. 2A). No major difference was observed between WT and Mettl3-cKO mice. Furthermore, the percentage of total macrophages (CD11b^+^F4/80^+^) in PerC was marginally decreased, probably due to the increased growth of ID8 cells (data not shown). Although the ID8 cells indicated active proliferating in the PerC, the ID8 cells induced a mild inflammatory response as there was not much immune cell recruitment observed (Fig. 2E A, 3B). However, the functional changes of these immune cells are obvious, as reflected by decreased M1 and increased M2 macrophage polarization leading to a low M1/M2 ratio (Fig. 2B-C). Additionally, it was revealed that gated on the CD11b^+^CD115^+^ population, the SPM cell population (ICAM2^−^MHC-II^hi^) constitutes the minority. In contrast, the LPM cell population (ICAM2^+^MHC-II^low^) constitutes most of the peritoneal tissue macrophages throughout the OC progression.

The literature suggests that TAM polarization is closely related to tumorigenesis and modulates the immune microenvironment by releasing various cytokines and chemokines [36]. Recently, it was demonstrated that transforming growth factor-beta-induced (TGFBI) protein and tenascin C (TNC) from ascites-derived TAMs promotes migration and progression of high-grade serous ovarian carcinoma (HGSC), characterized by a severe trans-coelomic tumor cell metastasis *via* the peritoneal fluid or malignant ascites [54]. OC patients with a high M1/M2 ratio of TAMs benefit from extended survival and an improved five-year prognosis [53, 55]. However, OC cells have indicated increased M2 phenotype polarization in vitro and in vivo [34, 35]. Consistently, the data of this study showed that OC cell implantation gradually polarizes PMs toward an M2 type. Both TAMs in PerC from WT and Mettl3-cKO mice exhibit a similar transition trend from M1 to M2 phenotype during OC progression, implying that METTL3 is dispensable for M1 to M2 differentiation during OC progression. Conversely, a recent study [37] suggested that *Mettl3* deficient mice indicated increased M1- and M2-like TAM in B16 and LLC tumor models. This discrepancy may be explained by differences in tumor type (solid vs. liquid) and tumor immune microenvironments.

MDSCs are a heterogeneous population that can suppress the immune response by inhibiting T-cell function. In OC patients, MDSCs infiltrate local TME and systemically promote tumor growth, invasion, and metastasis. It has been shown that CCL2-CCR2 axis and CXCL1/2-CXCR2 axis are the main pathways that recruit M-MDSCs and PMN-MDSCs, to tumor microenvironments, respectively. In OC patient, CCL2 is overexpressed in primary tumor cells [56–58] and its receptor, CCR2, has been observed in TAMs isolated from OC patients [59]. Elevated CXCL2 is also detected in OC patients [60]. In tumor-bearing mice, treatment of CXCR2 antagonist reduces MDSCs migration to the peritoneal cavity [60, 61]. The increased secretion of CCL2 and CXCL2 may have accounted for the enhanced migration of MDSCs in the peritoneal cavity of tumor-bearing METTL3-cKO mice. However, whether these retained monocytes play an anti-tumor or pro-tumor activity remains inconsistent. A recent study showed that ID8 injection in *Ccr2*^−/−^ and *Ccr2*^+/+^ mice indicated similar tumor growth, suggesting that deficiency of monocyte trafficking has no obvious effect on ID8 tumor growth [51]. Additionally, a key role of M-MDSCs was proposed in immune surveillance in the ID8-fLuc model [62]. Nevertheless, enhanced recruitment of Gr-1^+^ MDSCs was observed in this research, which may account for the accelerated OC cell growth in *Mettl3*-deficient mice compared to WT mice.

Inflammation is often associated with cancer initiation, progression, and metastasis. One of the major challenges in understanding the connection between inflammation and cancer is identifying the inflammatory response stimulus, the source and target of the inflammatory signals, and how this can contribute to tumor progression. IL-1 family cytokines are secreted from immune cells *via* unconventional pathways, which regulate inflammatory response through diverse mechanisms.

Emerging evidence suggests that IL-1β promotes tumor progression *via* various mechanism that stimulates tumor angiogenesis and recruitment of myeloid cells [59–61]. In LLC tumors, it has been shown that IL-1β release impairs the phenotype of tumor-infiltrating T cells and accumulation of intratumoral neutrophils and macrophages, resulting in an immunosuppressive TME [63–65]. Furthermore, a long-term clinical study indicated that treatment with neutralizing IL-1β-specific antibodies dose-dependently reduced lung cancer incidence and mortality in a large cohort of patients with atherosclerosis with a history of myocardial infarction [66]. Thus, it implies that anti-IL-1β treatment benefits anti-tumor therapy [67]. Whether inflammasome activation and pyroptosis (one of the cell death pathways) are involved in IL-1β secretion during OC progression requires further assessment.

Consistent with previously reported unchanged immune cell compositions, IL-1β, a potent pro-inflammatory cytokine, is induced slightly during the early phase (1–4 weeks) of tumor progression (Fig. 4A-B). Interestingly, this study (Fig. 4A-B) revealed that LPS pre-treatment is necessary for IL-1β secretion by macrophages in response to ID8 cells. *Mettl3* inhibition enhances IL-1β secretion (Fig. 4A) but not transcriptional expression (Fig. 4B) by macrophages. In BMDMs, ID8 cell lysate induced IL-1β mRNA without LPS stimulation (Fig. 4B), which can be explained by bioactive compounds in lysate that activate BMDMs to up-regulate related gene expression *via* different signaling pathways. Notebly, only live ID8 cells could enhance IL-1β secretion but not transcription by BMDMs after LPS stimulation, suggesting a direct cell-cell interaction pathway between ID8 tumor cells and BMDMs that boosts IL-1β secretion. Additionally, no IL-1β secretion was detected in the supernatants of LPS-stimulated ID8 cells, even though a moderate increase of IL-1β transcription was observed at LPS of ≤ 100 ng/ml (ELISA not shown and Fig. S5B), indicating IL-1β is secreted from BMDMs but not ID8 cells.

In general, IL-1 family cytokines (e.g., IL-1β) secretion is closely related to inflammasome activation, even though the involved secretory mechanisms remain poorly understood [43]. The active IL-1β secretion requires proteolytic maturation from its precursor (pro-IL-1β) by the caspase-1-mediated inflammasome activation, GSDMD-mediated membrane pore formation [43, 68, 69], and in a rare case, by other proteases, such as during high neutrophilic inflammation [65, 66]. To investigate whether inflammasome activation is associated with IL-1β secretion induced by live ID8 cells, BMDM cell lysates and supernatants under different culture conditions were collected and subjected to Western blot analysis to measure cytotoxicity. No cleavage of caspase-1 and GSDMD was detected, and LDH release was not enhanced in the BMDM cells cocultured with ID8 cells. It indicates that inflammasome activation is dispensable for OC inoculation-induced IL-1β secretion by BMDMs. These data concomitantly reveal that OC cells only trigger a mild inflammatory response to create an immunosuppressive microenvironment, thereby escaping immune surveillance.

## Conclusion

This study compared the ID8 tumor cell growth between WT and myeloid-specific *Mettl3* gene knockout mice and revealed enhanced OC cell growth in Mettl3-cKO mice. *Mettl3* depletion in myeloid cell lineage facilitates the recruitment of MDSCs. This research furnishes novel insights into METTL3-mediated m6A methylation that regulates host immune response against OC.

## Materials and methods

### Mice

*Mettl3*^*fl/+*^ mice were generated by micro-injection of two guided RNAs targeting exon 4 of the *Mettl3* gene and two cassettes carrying *LoxP* sequence into C57BL/6J zygotes using the CRISPR-Cas9 gene-editing system. Offspring with correct gene insertion were identified and then crossed to generate *Mettl3*^*fl/fl*^ mice. Further crossing with *Mettl3*^fl/fl^ mice generated conditional Mettl3-knockout mice (Mettl3-cKO mice) with myeloid-lineage specific Cre-recombinase expressing mice (*Lyz2-Cre*^*+/+*^). Offspring were genotyped based on the PCR, and females with designated genotypes were used for tumor inoculation experiments. The knockout efficacy of *Mettl3* in Mettl3-cKO mice was determined in BMDMs by Western blot, qRT-PCR and dot blot. All mouse strains were bred under specific pathogen-free conditions in Shenzhen People’s Hospital, the Second Clinical Medical College, and approved by IACUC of Jinan University.

### Cell cultures and in vitro cell proliferation experiments

ID8 cells (iCell Bioscience Inc, Shanghai) were cultured with DMEM medium (Gibco Inc.) supplemented with 10% FCS (Gibco Inc.) and 1% Penicillin-Streptomycin (P/S, 10,000 U/ml) in an atmosphere of 5% CO_2_ at 37 °C. ID8 cells were harvested in the log growth phase for tumor inoculation by trypsinization. The single-cell suspension was prepared by filtration with 70 μm cell strainers (Corning Inc.) followed by washing with cold DPBS (Gibco Inc.) twice and centrifugation at 350 × g at 4 °C for 5 min. Filtered ID8 cells were counted before inoculation.

To obtain BMDMs, bone marrow cells collected from mouse femurs and tibia were treated with red blood cell lysis buffer (Sigma-Aldrich, Cat#R7757) once and subsequently cultured in 10-cm Petri dishes (around five million cells per dish) with 10 ml of DMEM medium supplemented with 10% FCS, 1% P/S and 10 ng/ml M-CSF (Novoprotein, Cat#CB34) in an atmosphere of 5% CO_2_ at 37 °C. The culture medium was half changed with fresh conditioned complete DMEM medium with 10 ng/ml M-CSF on Day 4. Mature BMDMs were harvested on Day 7.

### m6A dot blot

Total RNA was extracted using TRIzol reagent (Vazyme Biotech Co.,Ltd, R411-01) according to the manufacturer’s instructions. RNA concentrations were quantified by NanoDrop 2000 (Thermo Scientific). For the dot blot, 300ng total RNA was dropped directly onto the Hybond-N + membrane (GE Healthcare, RPN3003B) and then crosslinked by ultraviolet irradiation. The membrane was blocked in 5% nonfat milk for 1 h and then incubated with an anti-m6A antibody (Ablconal Technology, China, A19841) overnight at 4 ℃. The membranes were washed extensively and incubated with goat anti-Rabbit IgG-HRP for 1 h at room temperature. After extensive wash, the membrane image was generated with the ECL detection system (Millipore, Cat#WBKLS500). In addition, methylene blue staining was applied to verify that equal amounts of RNA samples were loaded on the membrane.

### Cytotoxicity assay

For in vitro cytotoxicity assay, 1 × 10^6^ of BMDMs from either WT or Mettl3-cKO mice were first plated in a 6-well culture plate with or without LPS (10 ng/ml) priming overnight and then cocultured with live ID8 cells (1:1) or in the presence of different doses of ID8 cell lysate. The ID8 cell lysate was freshly prepared by repeat freezing (at -80 °C) and thawing (at 60 °C) of cells multiple times. Cell death was evaluated based on LDH release in tissue culture media by Roche Cytotoxicity Detection Kit (Roche Applied Science, Cat#11,644,793,001). The total RNA of cultured cells was collected and subjected to IL-1β transcriptional level analysis by qRT-PCR. In addition, total cell lysates were collected and subjected to Western blot analysis for NLRP3, caspase-1, and GSDMD expression.

### Mouse tumor experiments

Either 1 × 10^5^ or 5 × 10^5^ ID8 cells were injected intraperitoneally into WT mice and monitored for eight weeks to test the tumorigenesis of ID8 cells. The formation of the ascites was monitored, and the numbers of spheroids in the PerC were quantitated under the microscope. After successfully establishing the OC model, 5 × 10^5^ ID8 cells in 1 ml DPBS were injected intraperitoneally into each mouse for tumor cell injection on Day 0. Mice were sacrificed on weeks 1, 4, and 8 or 2, 5, and 9 of tumorigenesis. If the ascites were present, needles were first used to extract the peritoneal fluid. 5 ml DPBS containing 1% FBS, 1 mM EDTA, and 1% P/S was used for the peritoneal lavage of each mouse. Collected peritoneal lavage was centrifugated at 350 × g, 4 °C for 5 min. Cell pellets were treated with red blood cell lysis buffer 1–2 times if red blood cells were present. Subsequently, cell pellets were resuspended in the complete DMEM medium and were subjected to flow cytometry analysis, qRT-PCR, Western blot, H&E stain, and immunofluorescent staining. The supernatants of peritoneal lavage were collected for cytokines analysis. In some experiments, peripheral blood was collected from submandibular veins of tumor-bearing mice and allowed to clot for two hours at room temperature to acquire serum. The serum was stored at -80 °C for cytokines measurements by ELISA.

### Granulocytes isolation

Eight weeks after 5 × 10^5^ ID8 cells intraperitoneally injected into WT or Mettl3-cKO mice, Peritoneal lavage fluids were collected. Granulocytes were isolated using density gradiant centrifugation and separated with biotin-conjugated anti-mouse Gr-1^+^ beads. For naïve granulocytes isolation, bone marrow from the tibia of 12 weeks of WT and Mettl3-cKO mice was used. Isolated granulocytes were lysed with TRIzol reagent.

### Flow cytometry analysis

For FACS analysis of total PerC cells, 2 × 10^6^ cells were firstly stained for viability with Zombie Aqua Fixable Viability Kit (BioLegend, Cat#423,102). Next, cells were stained in 100 µL PBS with a 1:500 volume of antibody and incubated at room temperature for 15 min, protected from light. After incubation, cells were washed with cold FACS buffer (DPBS with 1% FCS) once again and subsequently stained for expression of surface markers. For surface marker staining, cells were stained with corresponding antibodies (volume ratio at 1:200) in 100 µL FACS buffer on ice for 30 min, protected from light. After incubation, cells were washed with cold FACS buffer twice and fixed with 4% PFA. For intracellular FACS staining, cells that have been done for surface staining were permeabilized with Foxp3/Transcription Factor Staining Buffer Set (Invitrogen, Cat#00-5523-00) and then stained with the corresponding antibody. Cells were acquired by BD Celesta and analyzed with Flowjo (Flowjo LLC, OR, USA) software.

### Immunofluorescent staining

Cell smears of total PerC cells were prepared by cytospin (350 × g, 5 min) and fixed with cold methanol for 5 min, followed by 4% PFA for 10 min at room temperature. Cells were then permeabilized with 0.1% Triton X-100 for 5 min at room temperature, followed by washing cell smears three times with 1× PBS. After permeabilization, the cell smears were washed three times with 1× PBS. Next, at room temperature, 5% BSA was used to block cells for 30 min. Next, cells were sequentially incubated with AF594-conjugated anti-CD68 antibody (1:250, BioLegend, Cat#137,020) for 2 h at room temperature and FITC-conjugated anti-Ki-67 antibody (1:100, eBioscience, Cat#11-5698-82) overnight at 4 °C. Finally, the cell smear was counterstained with DAPI (Sigma-Aldrich, Cat#D9542) for 5 min.

### ELISA

Cytokines levels in tissue culture conditioned supernatants, peritoneal lavage, and sera were measured using the following kits: IL-1β (Invitrogen, Cat#88-7013-88), IL-6 (R&D Systems, Cat#M6000B); IL-10 (R&D Systems, Cat#M1000B), IL-12 (R&D Systems, Cat#M1270), TNF-α (R&D Systems, Cat#MTA00B), CCL-2 (Absin, Cat#abs520016-96T), CXCL2 (Neobioscience, Cat#EMC122.96) according to instruction manuals of manufactures.

### Western blot

Cell lysates were separated by 10% or 12% SDS-PAGE, transferred to PVDF membranes, and probed with anti-NLRP3 antibody (CST, Cat#15101S), anti-β-actin antibody (CST, Cat#4970S), anti-GSDMD antibody (CST, Cat#39754S) and anti-caspase-1 antibody (AdipoGen, Cat#AG-20B-0042-C100). HRP-conjugated anti-Rabbit IgG antibody (Sigma, Cat#A0545) or HRP-conjugated anti-mouse IgG antibody (Invitrogen, Cat#31,430) was used as a secondary antibody. Immunoblots were developed using a chemiluminescent HRP-conjugated substrate (Millipore, Cat#WBKLS500) and imaged on a ChemiDoc™ Touch Imaging System (Bio-Rad Laboratories).

### H&E staining

Cell smears of total peritoneal cells were prepared by cytospin (350 × g, 5 min), dried, and fixed with 4% PFA. Cell smears were stained with hematoxylin for 5 min, then eosin for 30 s. Finally, cell smears were washed with 70% ethanol once and fixed with the mounting medium for imaging.

### Quantitative real-time PCR

The total PerC cells or cultured BMDMs were lysed with TRIzol reagent. The total RNA was extracted using UNlQ-10 Column Trizol Total RNA Isolation Kit (Sangon, Cat#B511321-0100) and reversely transcribed into cDNA using ReverTra Ace™ qRT-PCR Kit (TOYOBO, Cat#FSQ-101). cDNA was 1:10 diluted for qPCR measurements. Fold changes were calculated based on the 2^(–∆∆Ct)^ method or 2^(–∆Ct)^ method to reference genes. The primers for diverse genes are listed as follows:

*Mettl3* Forward: 5’-GGACACGTGGAGCTCTATCC-3’.

*Mettl3* Reverse: 5’-TGGGTTCCTTAAATCCAAGTGC-3’.

*Il1β* Forward: 5’-TGGACCTTCCAGGATGAGGACA-3’.

*Il1β* Reverse: 5’-GTTCATCTCGGAGCCTGTAGTG-3’.

*Hprt1* Forward: 5’-GCGTCGTGATTAGCGATGATG-3’.

*Hprt1* Reverse: 5’-CTCGAGCAAGTCTTTCAGTCC-3’.

*Cybb* Forward: 5’-TGTGGTTGGGGCTGAATGTC-3’.

*Cybb* Reverse: 5’-CTGAGAAAGGAGAGCAGATTTCG-3’.

*Actb* Forward: 5’- ATGACCCAAGCCGAGAAGG-3’.

*Actb* Reverse: 5’- CGGCCAAGTCTTAGAGTTGTTG-3’.

*Gapdh* Forward: 5’-AACGGGAAGCTCACTGGCATG-3’.

*Gapdh* Reverse: 5’-CCACCACCCTGTTGCTGTAG-3’.

### Single-cell RNA sequencing (scRNA-Seq) and data analysis

The single-cell RNA sequencing library was constructed with a 10× Genomics platform (Shanghai Jiayin Biotechnology Ltd., China). Briefly, cellular suspensions (10,000 cells) were loaded on the Chromium Controller (10× Genomics, Pleasanton) to generate Gel Bead-In-Emulsions (GEMs). Next, barcoded sequencing libraries were conducted following the instruction manual of the Chromium Single Cell 3’ Reagent Kits v3 (10× Genomics). The sequencing was performed following the library preparation with paired-end sequencing of 150 nt at each end on one lane of NovaSeq 6000 per sample.

CellRanger v5.0.1 software was applied to demultiplex the Illumina BCL output into FASTQ files. The Cell Ranger count was then applied to each FASTQ file to align reads to the GRCm38 (mm10) reference genome and generate barcode and unique molecular identifier counts. We followed the Seurat v3.2.0 integrated and comparative analysis workflows to do all scRNA-seq analyses [70]. For quality control and filtering out low-quality cells, only cells expressing more than 200 genes (defined as genes detected in at least three cells) and fewer than 20% mitochondrial genes were selected. As a result, 15,280 captured single cells (about 7600 cells per patient, WT:7504, CKO:7776) passed quality control for further batch correction and unbiased clustering.

The datasets were integrated based on ‘anchors’ identified between datasets (nfeatures = 2000, normalization.method=‘SCT’) before linear dimensional reduction by principal-component (PC) analysis. The top 30 PCs were included in a UMAP dimensionality reduction. After obtaining the top 30 PCs, we computed the shared nearest-neighbor graph and identified clusters in the network using the Louvain algorithm with a resolution of 0.6. The UMAP method (Uniform Manifold Approximation and Projection) was used to visualize unsupervised clustering. Differential gene expression or marker gene was determined by the ‘findMarkers’ function with the default Wilcoxon’s rank-sum test either as one versus the rest or as a direct comparison with parameters’ min.pct = 0.1’ and ‘logfFC threshold = 0.25’. Cell cluster identities were determined using known gene markers of individual cell types. We used the ‘clusterProfiler’ package for differential expression gene GO and KEGG pathway annotations and enrichment analysis.

### Statistics analysis

GraphPad Prism (GraphPad Software, San Diego, CA) was used for data processing. All data were expressed as Mean ± SD. An unpaired *t*-test was used to analyze most data, as illustrated in corresponding figure legends. Significance was set as * for *P* < 0.05, ** for *P* < 0.01, and *** for *P* < 0.001.

### Electronic supplementary material

Below is the link to the electronic supplementary material.

Supplementary Material 1

## Data Availability

The data supporting the conclusions of this article have been provided in this article and its additional files. In addition, all data from this study can be obtained from the corresponding author upon reasonable request.
