# Supplementary material for: METTL3-mediated m6A methylation regulates ovarian cancer progression by recruiting myeloid-derived suppressor cells
Source: Cell Biosci. 2023 Nov 6;13:202. doi: 10.1186/s13578-023-01149-6 (PMC10629157; doi:10.1186/s13578-023-01149-6)
Supplement: Supplementary file 1 — Supplementary Material 1 [file 13578_2023_1149_MOESM1_ESM.docx]

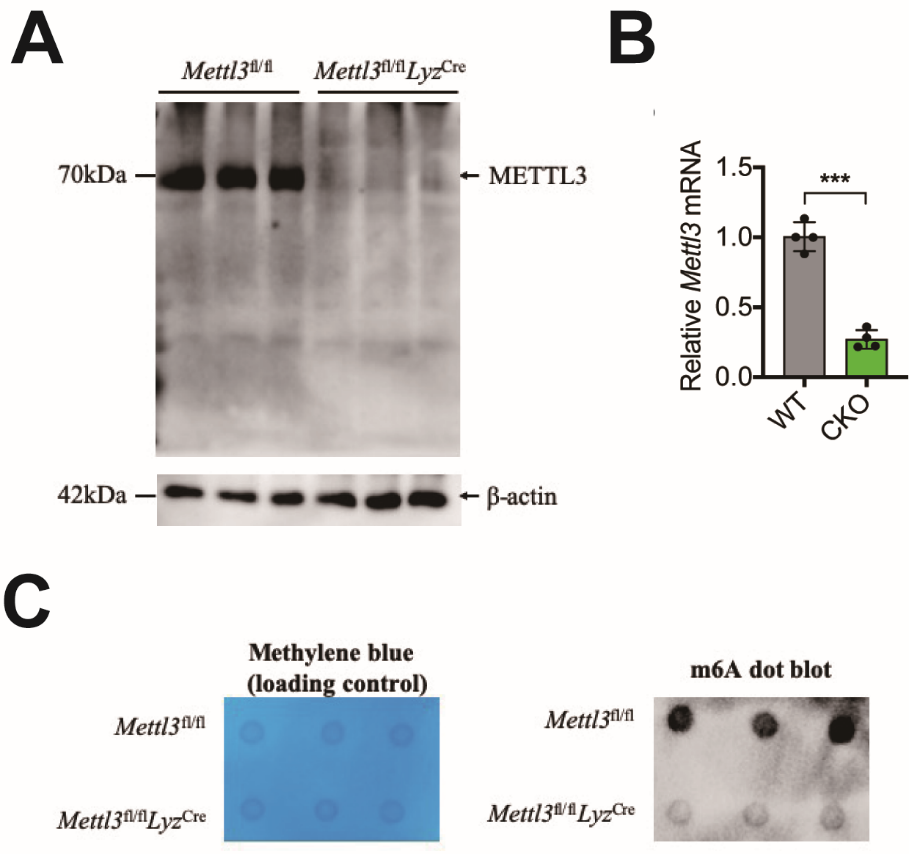


**Figure S1. METTL3 was significantly knocked down in BMDMs.** METTL3 expression level in BMDMs was detected by Western blot (A) and qRT-PCR (B), and GAPDH was used as the internal control. Anti-METTL3 antibody (Abcam, 195352, 1:1000) and anti-GAPDH antibody (CST 4970, 1:1000) were used. Bars represent SD; ****P* < 0.001. (C) Representative dot blot image of m6A of WT and Mettl3-cKO BMDMs. Anti-m6A antibody (Abclonal Techonology, China, A19841, 1:1000) was used. Methylene blue staining showed equal loading amount of total RNAs.


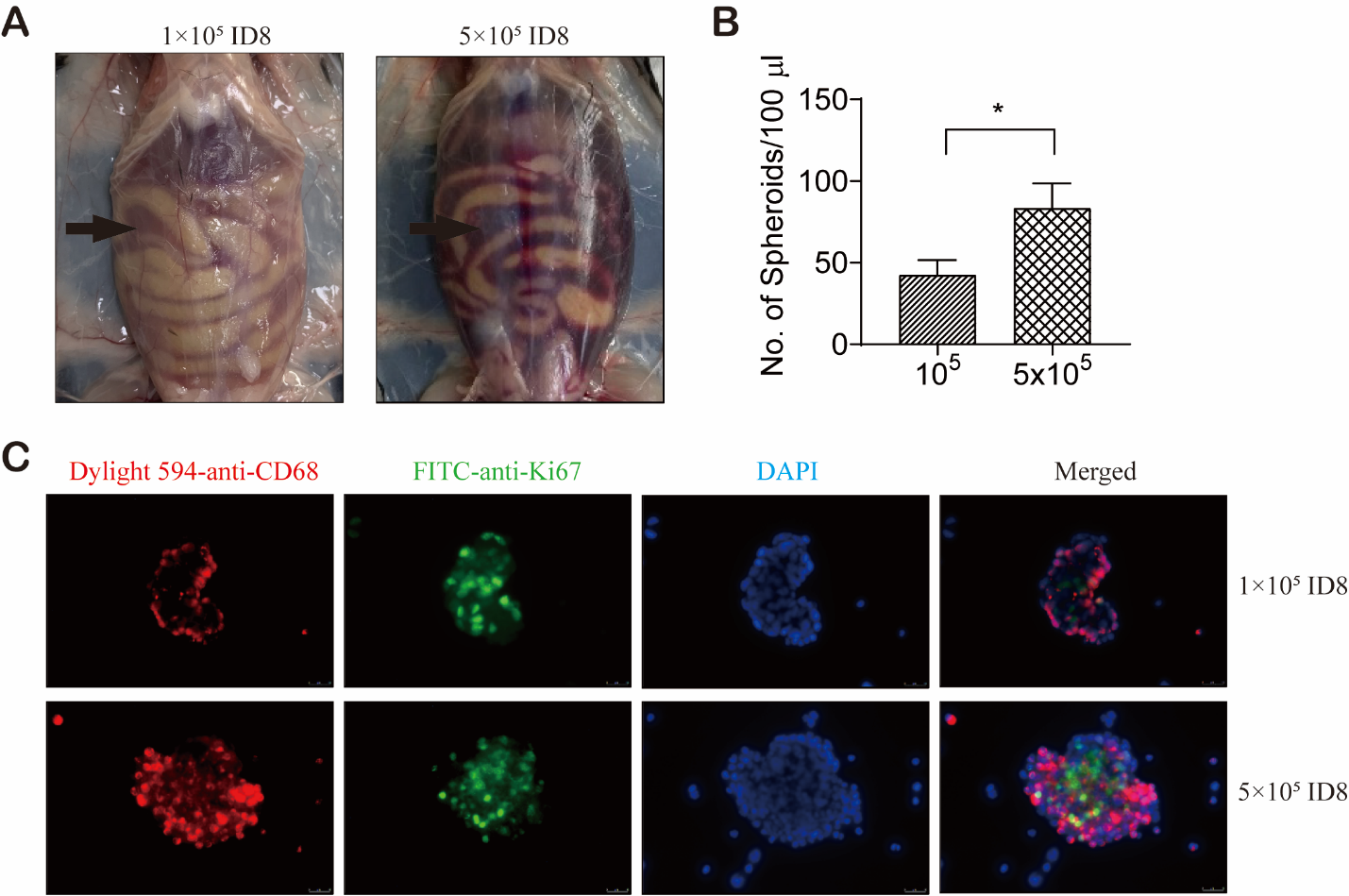


**Figure S2. Establishment of murine ID8 model.** 1×10^5^ or 5×10^5^ ID8 cells were injected intraperitoneally into C57BL/6J WT mice to test ID8 tumorigenesis. After eight weeks, gross appearances of the abdomen (A) and ascites formation (B) in the PerC are shown and quantitated. Bars represent SD; ****P* < 0.001. (C) Representative immunofluorescent images show proximity between CD68^+^ macrophages and Ki67^+^ ID8 tumor cells. DAPI is used for the visualization of the cell nucleus.


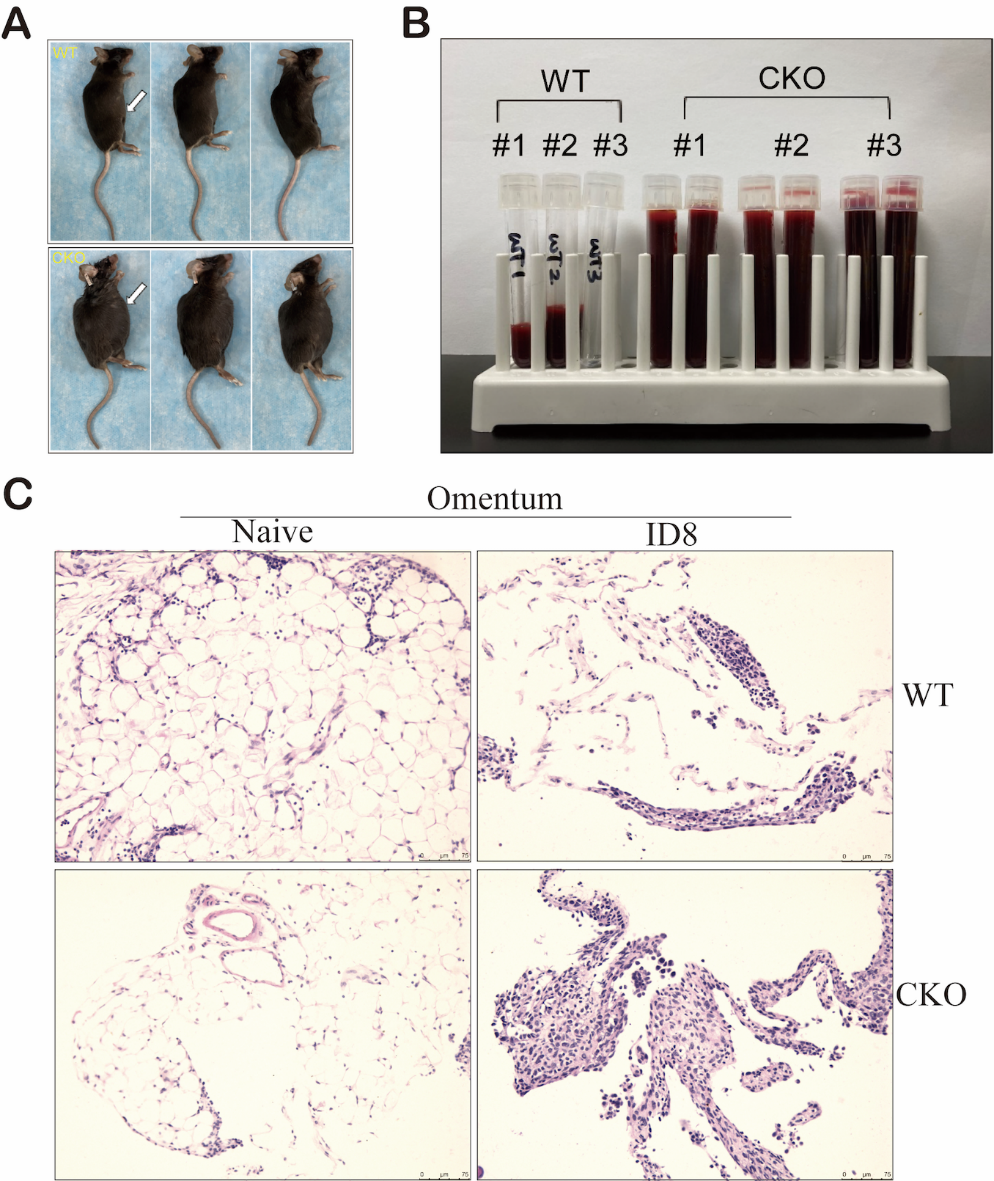


**Figure S3. Abdominal appearance and ascites formation in WT and Mettl3-cKO mice at the late stage of ID8 tumorigenesis.** (A) Representative images of mice's abdomen show much more severe swelling in Mettl3-cKO mice than in WT mice, and (B) A significantly higher volume of ascites extracted from Mettl3-cKO mice than WT mice at the late stage of tumorigenesis. (C) Histological images of omentum from both naïve and tumor-bearing WT and Mettl3-cKO mice. Scale bar = 75 μm.


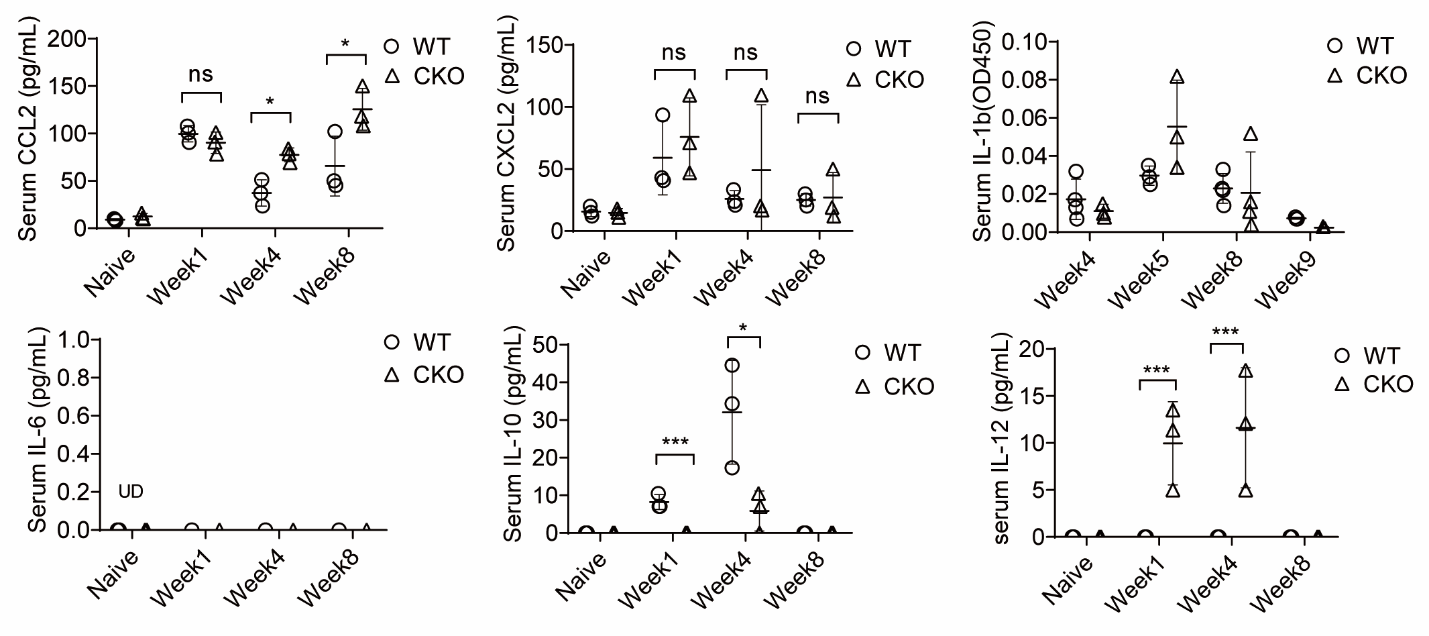


**Figure S4. Detection of cytokines and chemokines in peripheral blood.** Cytokines in the peripheral blood of mice sacrificed in Weeks 1, 4, and 8 were analyzed by ELISA. Mettl3-cKO mice have distinguishably higher IL-12 and CCL2 than WT mice. Corresponding to the ELISA data for peritoneal lavage, serum IL-10 is higher in WT mice than in Mettl3-cKO mice. There is no significant difference in IL-1β and CXCL2 between WT and Mettl3-cKO mice. IL-6 in serum is not detectable in all groups. Each dot represents one animal. Bars represent SD; **P* < 0.05, ***P* < 0.01, ****P* < 0.001.


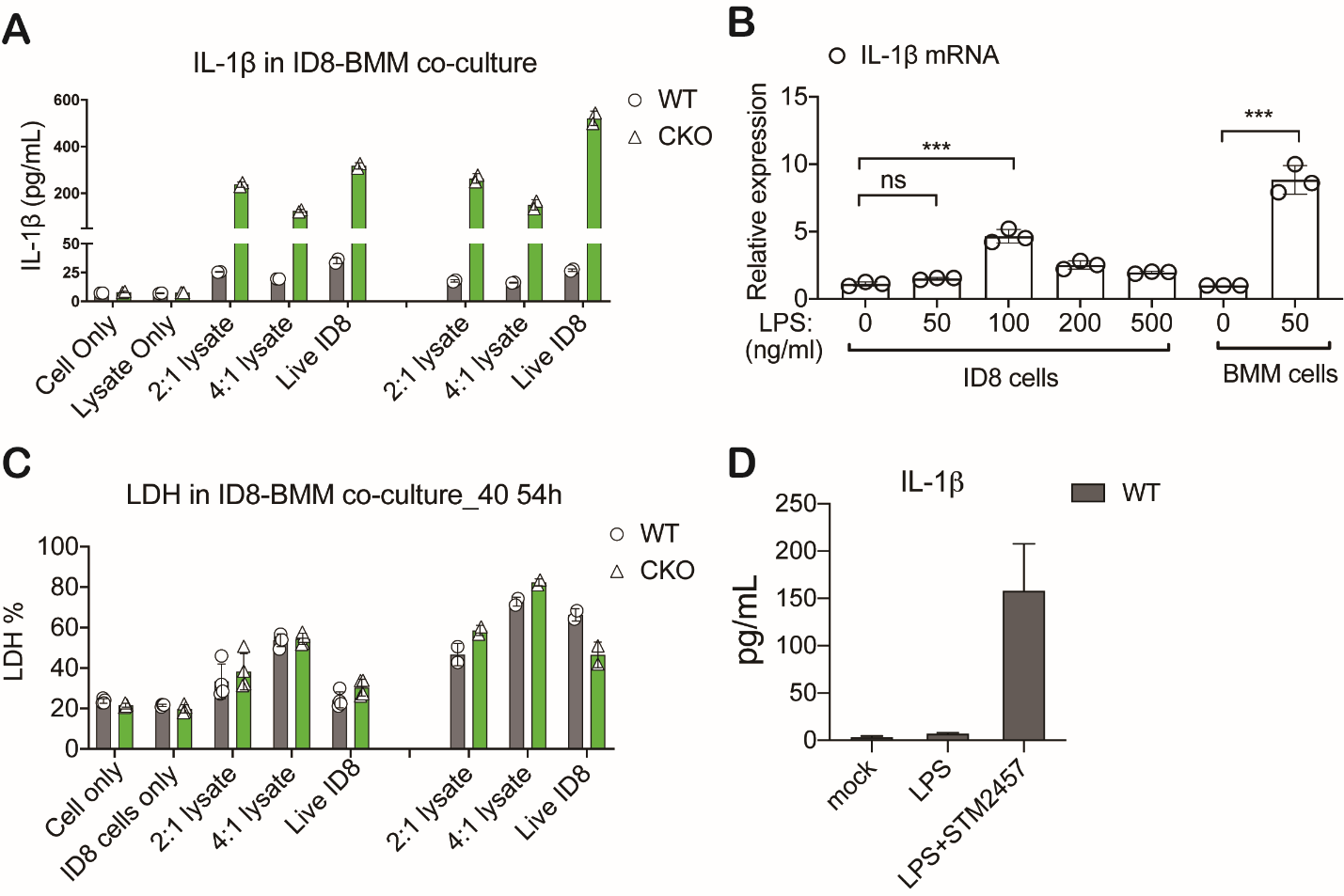


**Figure S5. Viable ID8 cells but not cell lysate enhance IL-**1**β secretion by macrophages and deletion of *Mettl3* increases IL-**1**β secretion.** (A) IL-1β secretion in the supernatants of ID8-BMDM coculture after 40 and 54 hours of incubation was detected by ELISA and LDH release assay. (B) Detection of mRNA transcription of IL-1β in ID8 cells and BMDMs with or without LPS treatment by qRT-PCR. (C) Cytotoxicity measurement by LDH release assay for samples as (A). (D) Wide-type BMDMs enhanced IL-1β secretion in the presence of a specific catalytic inhibitor of METTL3, STM2457 (20nM). Bars represent SD; ns, not significant, ****P* < 0.001.


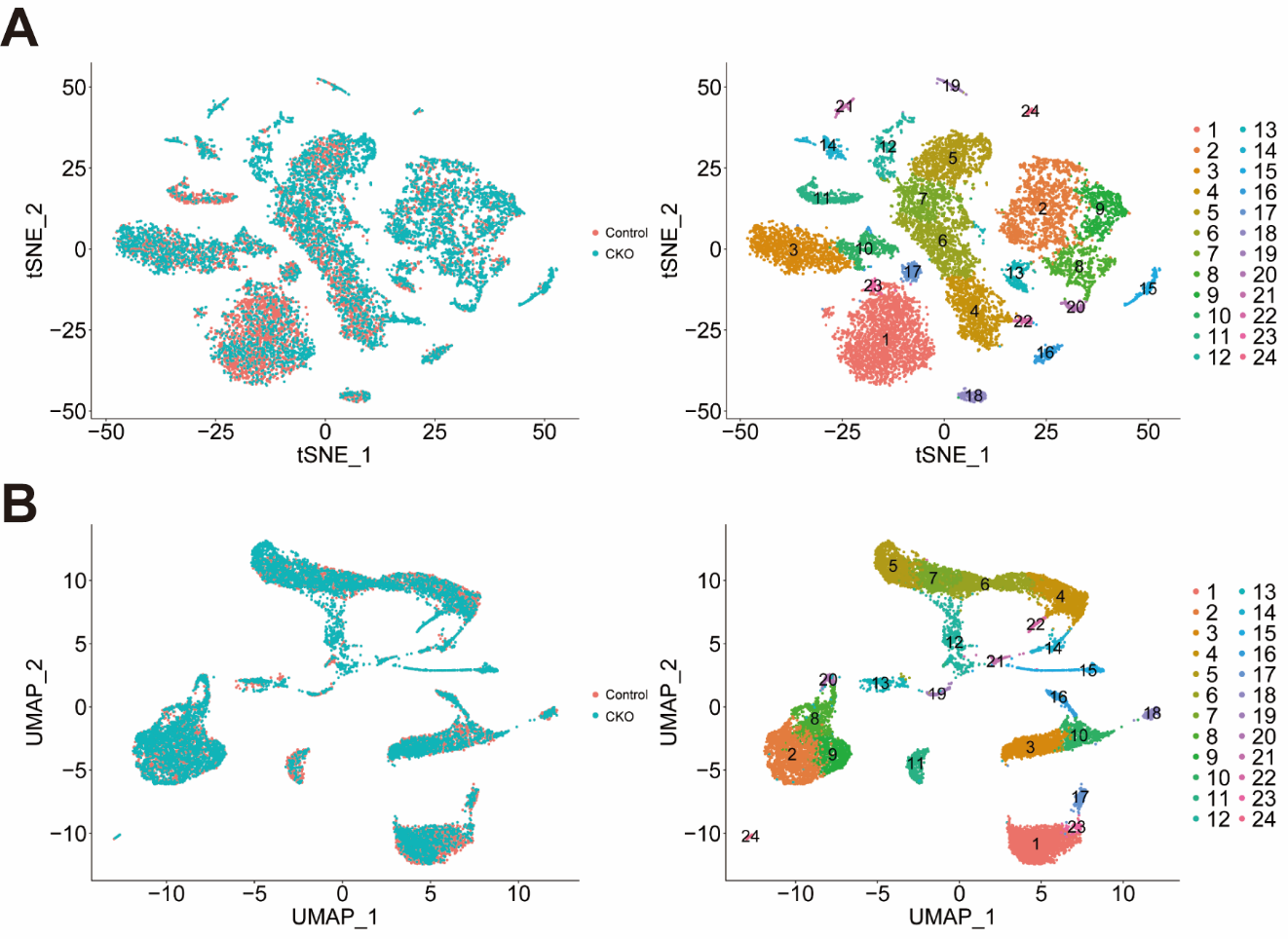


**Figure S6. A total of 24 distinct cell populations were clustered based on data from scSeq.** The aligned dataset of clustering was shown using (A) the "t-SNE1 vs. t-SNE2" method and (B) the "UMAP1 vs. UMAP2" method, respectively. Note: the left panel indicates the distribution of different samples. Red: WT (N11), Blue: CKO (N6); the right panel indicates clustering distribution after integrating two samples. Different colors represent different cell clusters.
